# Bloodletting in Purperal Eclampsia

**Published:** 1876-10

**Authors:** 


Bloodletting in Puerperal Eclampsia. Pathology and Therapeutics: The Old and the New. By Henry Fraser Campbell, M.D., of Augusta, Ga., Fellow of the American Gynæcological Society; Corresponding Member of the Imperial Academy of Medicine at St. Petersburg, Russia; and Professor of Operative Surgery and Gynæcology in the University of Georgia. (Reprint from the August Number of the *American Journal of Obstetrics.*) New York: Wm. Wood & Co. 1876.

In this pamphlet of forty-eight pages, Dr. Campbell reviews the opinions of pathologists on the subjects of this formidable difficulty of the puerperal state. While we have been very forcibly impressed with the theory of uræmic poison being an agent in the production of this malady, yet, with Dr. Campbell, we agree in believing that newly-discovered remedies are too apt to displace and drive from use those which the experience of ages and the philosophy of the present have sanctioned. Blood-letting—Dr. Campbell's sedative, Dr. Miller's prophylactic against fatal plethora of the brain, from the mechanical influence of the convulsion itself, and Dr. Johnson's *sine qua non* in the treatment of puerperal eclampsia—we cannot consent to ignore. What ever may be the prime agency in its production, the disease may be, as the author insists, the immediate result of irritation. The success claimed for the chloroform, opium, and blood-letting plans of treatment does not conflict with this theory.

The paper before us, in many respects, commands our admiration, and is evidently entitled to careful perusal.

